# Numb and Alzheimer’s Disease: The Current Picture

**DOI:** 10.3389/fnins.2012.00145

**Published:** 2012-10-04

**Authors:** Dimitrios Ntelios, Benedikt Berninger, Georgios Tzimagiorgis

**Affiliations:** ^1^Laboratory of Biological Chemistry, Medical School, Aristotle University of ThessalonikiThessaloniki, Greece; ^2^Department of Physiological Genomics, Institute of Physiology, Ludwig-Maximilians University MunichMunich, Germany

**Keywords:** Alzheimer’s disease, numb, neurogenesis, notch signaling

## Abstract

Twenty-three years ago, numb was identified as a critical regulator in *Drosophila* sensory organ precursor cell asymmetric divisions. Beyond the recently recognized role in carcinogenesis, Numb seems to be important in Alzheimer’s disease. This assertion comes from the involvement in various processes such as synapse morphogenesis, amyloid precursor protein trafficking, notch signaling, and neurogenesis. The purpose of the present mini-review is to provide the current picture of Numb’s participation in mechanisms underlying Alzheimer’s disease pathogenesis and emphasize potential aspects for future research.

## Introduction

Alzheimer’s disease (AD) is a serious debilitating neurodegenerative disorder described by the German psychiatrist and neuropathologist Alois Alzheimer in 1906 during a lecture in Tubingen. It is the most common form of dementia affecting millions of people worldwide. The major clinical manifestations are memory loss and cognitive decline. The primary neuropathological features of this disorder is neuronal cell death, synaptic loss, amyloid plaques, and neurofibrillary tangles with a unified theory explaining those findings a major unresolved issue. Amyloid plaques are extracellular deposits containing Aβ peptide, a proteolytic product derived from the amyloid precursor protein (APP). Mainly there are two modes of proteolysis: the sequential action of β and γ secretase which generates the Aβ peptide (amyloidogenic mode) and the α and γ secretase pathway (non-amyloidogenic mode). Neurofibrillary tangles are aggregates of hyperphosphorylated tau, a microtubule associated protein (Selkoe, 2001). A very important milestone in this century of AD research was the identification of presenilin 1 coding gene *PSEN1* as a familiar AD cause (Sherrington et al., 1995). In this study *Drosophila* *numb* human homolog was mapped due to its location 50 kb away from *PSEN1*. Since then, there are many indications from the published literature that Numb may play a role in Alzheimer’s disease pathogenesis and progression.

## Numb Overview

Initially numb was described as a cell fate determinant during sensory organ development in *Drosophila* embryos. Its name eloquently describes the sensory neuron depletion caused by *numb* loss of function mutations (Uemura et al., 1989). In humans there are two homologous genes, *NUMB* and *NUMB-Like*. *NUMB*, located in 14q23, consists of 13 exons and by alternative splicing produces at least nine different transcripts (Haider et al., 2011). The mRNA binding protein Musashi-1 (Kawahara et al., 2008) and miR146a (Kuang et al., 2009) downregulate Numb protein levels. Numb does not have any enzymatic activity instead with its protein interaction domains acts as a molecular scaffold. Adhesion molecules, kinases, endocytic proteins, and ubiquitin ligases are the Numb’s interacting network partners and reflect its multiple physiological roles (Gulino et al., 2010). Numb associates with membranes and the subcellular localization is regulated by G-coupled receptors (Dho et al., 2006) and several kinases among them aPKC (Smith et al., 2007), Ca2+/calmodulin-dependent protein kinases (Tokumitsu et al., 2005), AAK1 (Sorensen and Conner, 2008). At last, conjugation of ubiquitin by LNX (Nie et al., 2002), Siah-1 (Susini et al., 2001), Mdm2 (Colaluca et al., 2008) targets Numb for proteasomal degradation.

## Numb Endocytosis and APP Trafficking

**Figure 1:**
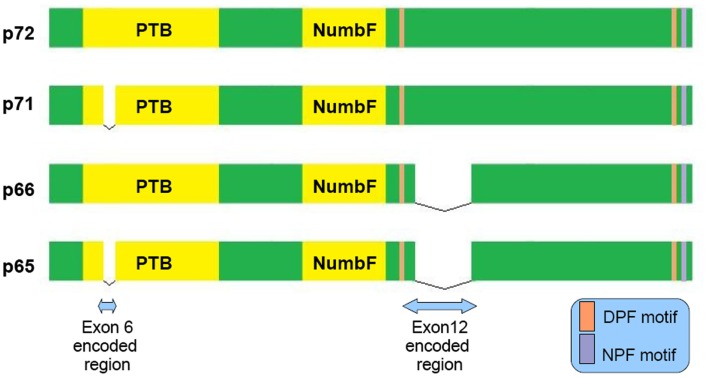
**Schematic representation of the four main Numb isoforms and their domain structure**. PTB (Phosphotyrosine Binding Domain), NumbF (Numb family of proteins domain), NPF (asparagine-proline-phenylalanine), DPF (aspartate-proline-phenylalanine).

Numb is a clathrin-associated sorting protein (CLASP) recognizing the [FY]XNPX[YF] motif (X any aminoacid; Traub, 2009). By its DPF and NPF motif (see Figure 1) binds to AP-2 subunit α-adaptin (Santolini et al., 2000) and EPS15 (Salcini et al., 1997) accordingly, well known constituents of the endocytic machinery. It acts not only in the clathrin mediated endocytosis, but also in the Arf6 pathway and to the endocytic recycling through its interaction with EDH1 and EHD4 proteins (Smith et al., 2004). Different molecules like: L1 (Nishimura et al., 2003), integrin-b (Nishimura and Kaibuchi, 2007), E-cadherin (Kuo et al., 2006; Rasin et al., 2007; Sato et al., 2011) require numb for their endocytosis and proper membrane targeting.

**Figure 2:**
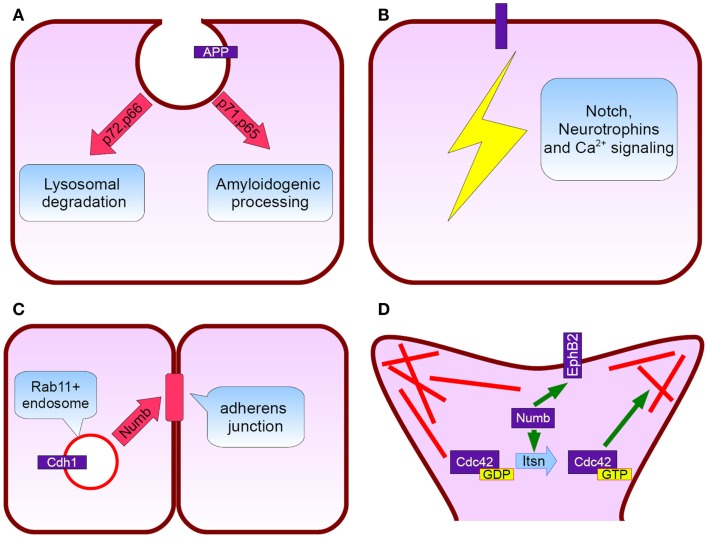
**Numb protein functions relevant to Alzheimer’s disease**. **(A)** APP subcellular trafficking: p72 and p66 isoforms (exon 6+) promote APP rooting to lysosomes whereas p71 and p65 (exon 6−) APP amyloidogenic processing **(B)** Notch, neurotrophins, and calcium signaling pathways fine tuning, **(C)** Cadherin 1 targeting to adherens junctions and subsequently neurepithelium architecture preservation, **(D)** Numb interacts directly with EphB2 and additionally induces Intersectin’s GTP for GDP exchange action on Cdc42. Cdc42 regulate spine dynamics by local actin remodeling (red lines).

In AD neurons endocytosis is impaired, with the detection of enlarged endosomes as an early neuropathological finding (Cataldo et al., 1997). Interestingly, approximately 70% of the Aβ peptide secreted to the interstitial fluid is generated by endocytosis related mechanisms (Cirrito et al., 2008). Because α secretase predominantly localizes to the cellular membrane whereas β secretase to acidic intracellular compartments (endosomes), APP’s intracellular trafficking has a principal role in amyloidogenesis (Small and Gandy, 2006). There is evidence that Numb may serve as a modulator of APP sorting. Based on FRET and immunoprecipitation experiments, a direct interaction between Numb and APP cytoplasmic tail through Numb phosphotyrosine binding domain (PTB) is established (Roncarati et al., 2002). Numb PTB domain recognizes the YENTPY conserved motif in APP cytoplasmic tail (Roncarati et al., 2002). The affinity of these interaction depends upon the Numb isoform examined and APP intracellular domain phosphorylation status (Tamayev et al., 2009). According to those *in vitro* data the increased Thr668 and Tyr682 phosphorylation in AD patients makes this interaction weaker (Lee et al., 2003). Also the p71 isoform (see Figure 1) docks with a greater affinity than the p72 (Tamayev et al., 2009). Many molecules comprising the APP intracellular part interactome are PTB domain containing proteins. Characteristic examples are Fe65, JIP, X11α, and β, whose role in AD has attracted researchers interest (King and Turner, 2004). Undoubtedly all those factors consist a fine tuned protein network and alterations could have negative or positive implications, e.g., X11α slows APP processing reducing Aβ deposition (King et al., 2003).

*NUMB* exon 6 alternative splicing give rise to isoforms that differentially affect APP processing. Exon 6 encodes a 11 aminoacid sequence in Numb PTB domain. Human exon 6− isoforms overexpression increase Aβ production in SH-SY5Y cells bearing the Swedish double mutation K595N/M596L in APP. On the other hand exon 6+ isoforms lead to lower Aβ production than Numb wild type cells. This result is attributed to different subcellular APP trafficking. Exon 6+ isoforms induce higher APP routing to lysosomes for degradation whereas exon 6− favor APP recycling (Kyriazis et al., 2008). Additionally Numb-like promote APP amyloidogenic processing (Schobel et al., 2006). In cortical cell cultures treated with Aβ, exon 6− isoforms are upregulated (Chigurupati et al., 2011). Interestingly, the same study reports increased Numb exon 6− protein isoforms in triple transgenic AD mouse model and parietal cortex from affected individuals. An important open question is the exact numb levels in the hippocampus from AD patients (an area great interest in AD) due to the elevated Musashi-1 (rna binding protein downregulating numb mrna) reported elsewhere (Lovell and Markesbery, 2005). In contrast to AD, reduced numb levels correlated with cancer development as shown in breast (Pece et al., 2004) and lung carcinomas (Westhoff et al., 2009). Whether Numb levels provide a biological explanation of why AD patients have lower incidence of malignancies than age-matched controls, remains to be clarified (Roe et al., 2010).

## Numb and Neurogenesis

In adult life nerve cells are continually born in the subgranular zone of the dentate gyrus and the subventricular zone (Curtis et al., 2011). Studies in humans and familiar Alzheimer’s disease mouse models, despite some controversies, demonstrated alterations in neurogenesis (Lazarov and Marr, 2010). Numb is essential for the asymmetric divisions during *Drosophila* embryo peripheral and central nervous system development. Due to its ability to interact with dividing cell’s polarity mechanisms, has the potential to distribute asymmetrically between the two daughter cells thereby enabling them to choose a different cell fate, acting as a binary switch (Zhong, 2008). On the contrary, there is an open discussion about the exact role in cell fate determination during asymmetric cell divisions in mammals. However, it is evident that numb plays critical role during mammalian neurogenesis also. Several Numb/Numbl conditional double knockout mice demonstrated severe defects in brain development (Petersen et al., 2002, 2004; Li et al., 2003). It is proposed that Numb is crucial for the neurepithelium architecture maintenance. It associates with Rab11+ recycling endosomes containing Cadherin 1 and is essential for the correct membrane targeting of this adherens junction component (Rasin et al., 2007). Postnatal Numb/numbl deletion seriously compromises ependymal wall integrity and subventricular zone homeostasis (Kuo et al., 2006). Despite the recent data indicating that SVZ neurogenesis largely stops after 18 months of age (Sanai et al., 2011) there are reports that stroke (Marti-Fabregas et al., 2010) and Huntington disease induce a substantial upregulation (Curtis et al., 2003). Furthermore, numb modulates signaling pathways such as Hedgehog (Marcotullio et al., 2006) and Notch with well recognized role in adult neurogenesis (Lai et al., 2003; Imayoshi et al., 2010).

## Numb and Synapse Morphogenesis

A very interesting observation in AD is the dramatic reduction in dendritic spines and its good correlation with disease progression (Terry et al., 1991). Cortical rewiring through new synapse formation is a key mechanism for memory and cognition (McAllister, 2007). Numb affects synapse formation through several mechanisms. In particular, Numb p72 isoform overexpression in cultured hippocampal neurons leads to an increase in spine length. In the case of aminoacid 1–183 and 184–592 fragment overexpression, an additional decrease in spine density and percentage of protrusions with a spine head is observed. Numb interacts with intersectin [possesses a guanine nucleotide exchange factor (GEF) activity for Cdc42], Rac GEF Tiam1, and dual Rho GEF Kalirin. Cdc42, Rac, and Rho are members of the Rho family GTPases (Nishimura et al., 2006). They regulate actin dynamics and have a pivotal role in synapse formation as reflected from the mental retardation syndromes linked to genes participating in this signaling pathway (Ramakers, 2002). GEFs activate Rho proteins by a GTP for GDP exchange. In the case of intersectin we have to mention that is a chromosome 21 encoded protein with a direct involvement in Down syndrome (Chang and Min, 2009). At last, Numb interact with NMDA glutamate receptors and EphB2 (Nishimura et al., 2006). EphB2 is essential for the synapse to function properly (Lai and Ip, 2009) and is downregulated in AD brains (Cisse et al., 2011).

## Numb and Signaling Pathways

Much work has focused on the regulation of intracellular signaling cascades by Numb. First of all, Numb is the classical Notch pathway inhibitor. Mammals have four different Notch receptors mediating cell–cell communication (Brou, 2009). In adult nervous system, Notch signaling has linked with various functions from long term potentiation (Wang et al., 2004), to dendrite arborization (Berezovska et al., 1999) and neurogenesis (Breunig et al., 2007; Imayoshi et al., 2010). The p66 isoform was shown to promote Notch 1 ubiquitination by recruiting E3 ubiquitin ligase Itch (McGill and McGlade, 2003; McGill et al., 2009). The ability to exert a repressive effect on Notch signaling differs between Numb isoforms (Beres et al., 2011). It is proposed that AICD (APP’s C terminal part generated from γ cleavage) binds Numb and inhibits Notch (Roncarati et al., 2002). In the case of Notch 1 and 2 receptors, a direct interaction with APP was observed (Chen et al., 2006). Interestingly, Notch 1 immunoreactivity in the hippocampus of AD patients was increased (Berezovska et al., 1998). Similarly to APP, Notch receptors are α and γ secretase substrates with this proteolytic events being prerequisite for signal transduction. This is why, in the field of AD therapeutics, the main obstacle toward a safe γ secretase inhibitor is the compromised Notch signaling related toxicities (Oehlrich et al., 2011).

Beyond Notch, Numb seems to play a role in neurotrophin signaling. This pathway promotes neuronal survival (Heumann, 1994) and is perturbed in AD brains (Williams et al., 2006). Especially the TrkA and TrkB receptors are downregulated (Ginsberg et al., 2006). Recently a study demonstrated that Numb required for TrkB endocytosis and migration of cerebellum granule cells across BDNF gradient (Zhou et al., 2011). PC12 cell overexpressing exon 6− isoforms display increased NGF induced neurite outgrowth partly due to increased TrkA levels (Pedersen et al., 2002). This outgrowth is a well recognized phenomenon which makes PC12 cells a popular neuronal model. Furthermore TrkA and TrkB endocytosis in sympathetic and hippocampal neurons requires EHD4 (Pincher), a Numb binding partner (Valdez et al., 2005).

Several lines of evidence point toward a contribution to the calcium mishandling observed in AD (Supnet and Bezprozvanny, 2010). Exon 6− isoforms overexpression in PC12 cells induce neurite outgrowth without requiring NGF treatment in a way depending upon voltage-gated calcium channels (Lu et al., 2009). Additionally these cells are more vulnerable to Aβ toxicity due to a calcium related mechanism (Chan et al., 2002) and after trophic factor withdrawal from the culture medium TRPV6 is upregulated leading to calcium dysregulation and increased cell death (Kyriazis et al., 2010).

Recently, an interesting finding was that Numb interacts with the cell cycle regulatory protein Polo-like kinase 1 (Plk1; Schmit et al., 2012). Plk1 activity is elevated in AD patients and PLK1 silencing reduces Aβ-induced neuronal cell death (Song et al., 2011). Indeed, a link between cell cycle disturbances and neuronal death has now been established in AD. It is proposed that inappropriate reentry into a mitotic cell cycle triggers neuronal cell death (Herrup, 2012). In this context, cyclin-dependent kinase 5, which prevent this reentry (Zhang et al., 2010) regulates collapsin response mediator protein-2 (CRMP-2). Of note, CRMP-2 has a role in Numb-mediated endocytosis (Nishimura et al., 2003), is phosphorylated early in AD (Cole et al., 2007) and is a substrate for glycogen synthase kinase 3 (Gsk3β; Cole et al., 2004), a known molecular player in AD (Kremer et al., 2011).

## Conclusion

There is already ample *in vitro* evidence that this protein affect neurodegeneration in multiple ways. *In vivo*, Numb exon 6− isoforms has been shown to increase in both triple transgenic AD mouse model and affected human individuals. In the future the development of mice bearing a known AD causing mutation with targeted disruption of specific Numb isoforms will provide a unique angle to approach the study of Numb’s role in AD. To what extent AD pathogenic pathways are Numb-dependent *in vivo* is not yet clear but considering the data provided so far there are many exciting findings to come in the next years. The clinical significance of this molecule as a potential drug target or biomarker remains to be convincingly demonstrated by future studies. The fact that Numb has a tumor suppressor activity, makes problematic target for future drug research. However elucidating further the physiological relevance of this Numb APP interaction could provide us with a more complete understanding of the mechanisms involved in this devastating illness.

## Conflict of Interest Statement

The authors declare that the research was conducted in the absence of any commercial or financial relationships that could be construed as a potential conflict of interest.
